# Human adipose tissue H3K4me3 histone mark in adipogenic, lipid metabolism and inflammatory genes is positively associated with BMI and HOMA-IR

**DOI:** 10.1371/journal.pone.0215083

**Published:** 2019-04-08

**Authors:** Daniel Castellano-Castillo, Pierre-Damien Denechaud, Lluis Fajas, Isabel Moreno-Indias, Wilfredo Oliva-Olivera, Francisco Tinahones, María Isabel Queipo-Ortuño, Fernando Cardona

**Affiliations:** 1 Unidad de Gestión Clínica de Endocrinología y Nutrición del Hospital Virgen de la Victoria, Instituto de Investigación Biomédica de Málaga (IBIMA), Universidad de Málaga, Málaga, Spain; 2 Centro de Investigación Biomédica en Red de Fisiopatología de la Obesidad y la Nutrición, CIBERobn, Madrid, Spain; 3 Center for Integrative Genomics, University of Lausanne, Lausanne, Switzerland; 4 Department of Physiology, University of Lausanne, Lausanne, Switzerland; 5 Institut des Maladies Métaboliques et Cardiovasculaires, Inserm UMR 1048, Toulouse, France; 6 Unidad de Gestión Clínica Intercentro de Oncología Médica del Hospital Virgen de la Victoria, Instituto de Investigación Biomédica de Málaga (IBIMA), Universidad de Málaga, Málaga, Spain; Medical University Innsbruck, AUSTRIA

## Abstract

**Introduction:**

Adipose tissue is considered an important metabolic tissue, in charge of energy storage as well as being able to act in systemic homeostasis and inflammation. Epigenetics involves a series of factors that are important for gene regulation or for chromatin structure, mostly DNA methylation and histone-tail modifications, which can be modified by environmental conditions (nutrition, lifestyle, smoking…). Since metabolic diseases like obesity and diabetes are closely related to lifestyle and nutrition, epigenetic deregulation could play an important role in the onset of these diseases and vice versa. However, little is known about histone marks in human adipose tissue. In a previous work, we developed a protocol for chromatin immunoprecipitation (ChIP) of frozen human adipose tissue. By using this method, this study investigates, for the first time, the H3K4 trimethylation (H3K4me3) mark (open chromatin) on the promoter of several factors involved in adipogenesis, lipid metabolism and inflammation in visceral adipose tissue (VAT) from human subjects with different degrees of body mass index (BMI) and metabolic disease.

**Methodology:**

VAT was collected and frozen at -80°C. 100 mg VAT samples were fixed in 0.5% formaldehyde and homogenized. After sonication, the sheared chromatin was immune-precipitated with an anti-H3K4me3 antibody linked to magnetic beads and purified. H3K4me3 enrichment was analyzed by qPCR for LEP, LPL, SREBF2, SCD1, PPARG, IL6, TNF and E2F1 promoters. mRNA extraction on the same samples was performed to quantify gene expression of these genes.

**Results:**

H3K4me3 was enriched at the promoter of E2F1, LPL, SREBF2, SCD1, PPARG and IL6 in lean normoglycemic compared to morbid obese subjects with prediabetes. Accordingly H3K4me3 mark enrichment at E2F1, LPL, SREBF2, SCD1, PPARG and IL6 promoters was positively correlated with the BMI and the HOMA-IR. Regression analysis showed a strong relationship between the BMI with H3K4me3 at the promoter of E2F1 and LPL, and with mRNA levels of LEP and SCD. In the case of HOMA-IR, the regression analysis showed associations with H3K4me3 enrichment at the promoter of SCD1 and IL6, and with the mRNA of LEP and SCD1. Moreover H3K4me3 at the E2F1 promoter was positively associated to E2F1 mRNA levels.

**Conclusions:**

H3K4me3 enrichment in the promoter of LEP, LPL, SREBF2, SCD1, PPARG, IL6, TNF and E2F1 is directly associated with increasing BMI and metabolic deterioration. The H3k4me3 mark could be regulating E3F1 mRNA levels in adipose tissue, while no associations between the promoter enrichment of this mark and mRNA levels existed for the other genes studied.

## Introduction

Obesity and related disorders have become one of the greatest health problems in developed countries. Obesity usually involves an increased risk of metabolic syndrome (MetS), insulin resistance, diabetes, cardiovascular failure, stroke and some sort of cancers [1].

Adipose tissue, which plays an important role as a lipid storage tissue, has emerged as an active tissue that can produce a multitude of signaling molecules (cytokines, adipokines, lipids, …) affecting whole body function and participating in the metabolic disruption observed during obesity [2]. In addition to its role in metabolic homeostasis, the endocrine-related activity of this tissue is being currently studied in the context of a large number of diseases like diabetes, MetS, cardiovascular disease or cancer [3,4]. Indeed, obesity is accompanied by an increment in fat mass proportion, associated with adipose tissue dysfunction and increased inflammation [3,5]. Moreover, a limited lipid accumulation capacity of adipose tissue can provoke ectopic lipid accumulation, as muscle and liver lipid accumulation, which can result in insulin resistance, and eventually in type 2 diabetes mellitus [6].

A strong environmental component exists in obesity and related diseases. In this sense, epigenetics has been postulated to be an important regulation landscape in this etiology as environment factors like lifestyle, smoking or diet have been proposed to modify epigenetic regulation [7]. Nevertheless, the role of adipose tissue epigenetic modifications in the etiology of obesity and metabolic disorders has been usually restricted to DNA methylation studies [8]. Adipose tissue chromatin immunoprecipitation (ChIP) for histone marks has been carried out mostly in mice, and especially *in vitro* in adipose-derived mesenchymal stem cells. Probably due to difficulties found in human adipose tissue manipulation, no studies have investigated (to our knowledge) direct histone modifications in human adipose tissue [8].

In a previous work, we developed a ChIP method to determine H3K4me3 chromatin immunoprecipitation in small pieces of frozen human adipose tissue [8]. In this study, we applied this method to analyze histone methylations in adipose tissue of a cohort of 39 patients with different metabolic profiles. We analyzed promoter H3K4me3 levels and the gene expression of specific genes related to lipid metabolism, adipogenesis and inflammation, such as LEP, LPL, SREBF2, SCD1, PPARG, IL6, TNF and E2F1.

## Methodology

### Study population

This study was undertaken in 39 subjects, classified according to their BMI and glucose state as Lean Normoglycemic (Lean NG) (BMI<25 and glucose<100 mg/dl), Morbid Obese Normoglycemic (MO NG) (BMI>40 and glucose<100 mg/dl) and Morbid Obese Prediabetic (MO PD) (BMI>40 and glucose ranging ≥100 to <125). Study procedures included a comprehensive physical examination and blood analysis. The metabolic parameters for the 3 groups are presented in Table 1.

**Table 1:** Anthropometric and biochemical variables for each study group. Different letters mean significant differences between groups (p<0.05).

	Lean NG (n = 10)	MO NG (n = 10)	MO PD (n = 9)
**Age (years)**	54.40±13.93	40.50±8.34	47.11±8.28
**Gender (male/female)**	4/6	3/7	3/6
**BMI (kg/m^2^)**	22,91±1.52**a**	50,70±8.78**b**	56.12±7.99**b**
**Waist (cm)**	86.70±8.74**a**	134.70±20.27**b**	142.74±14.41**b**
**Glucose (mg/dl)**	91.10±6.04**a**	90,90±4.95**a**	111.89±4.37**b**
**Insulin (pmol/L)**	6.02±3.13**a**	20.26±15.61**b**	22.29±9.10**b**
**HOMA-IR**	1,34±0.69**a**	4.61±3.53**b**	6.14±2.48**b**
**Cholesterol (mg/dl)**	226.30±56.60	183.10±48.66	190.44±25.48
**HDL-C (mg/dl)**	56.20±16.81**a**	41.20±9.37**a,b**	39.67±5.54**b**
**LDL-C (mg/dl)**	144.46±45.19	120.84±45.76	120.16±22.17
**Tg (mg/dl)**	128.40±69.38	93.99±31.09	159.53±60.84
**SBP (mm Hg)**	129.90±25.00	135.44±30.73	141.29±15.15
**DBP (mm Hg)**	80.80±9.36	80.78±13.04	86.86±9.22

Body mass index (BMI); Homeostatic model assessment of insulin resistance (HOMA-IR); High-density lipoprotein cholesterol (HDL-C); Low-density lipoprotein cholesterol (LDL-C); Triglycerides (Tg); Postprandial triglycerides (Tg Post); Systolic blood pressure (SBP); Diastolic blood pressure (DBP).

Study subjects were recruited during 2012–2014 from patients that had undergone laparoscopic surgery for elective cholecystectomy, hiatal-hernia surgery, or bariatric surgery. Exclusion criteria included the presence of cardiovascular disease, arthritis, acute inflammatory disease, infectious disease, renal disease or patients receiving drugs that could alter the lipid profile or MetS parameters at the time of study inclusion. Smoking habits and alcohol consumption were measured using a standardized questionnaire.

The study was conducted in accordance with the guidelines laid down in the Declaration of Helsinki. All participants gave their written informed consent and the study was reviewed and approved by the Ethics and Research Committee of Virgen de la Victoria Hospital.

### Laboratory measurements

Serum parameters were measured following previous procedures [9]. Leptin and adiponectin were analyzed by enzyme immunoassay (ELISA) kits (DSL, Webster, TX, and DRG Diagnostics GmbH, Germany, respectively).

### Visceral adipose tissue RNA isolation

VAT was obtained during laparoscopic surgery. Biopsy samples were washed in physiological saline buffer and immediately frozen in liquid nitrogen. Biopsy samples were maintained at −80°C until analysis.

### Real-time quantitative PCR

RNA extraction and qPCR analysis was performed as described previously [9]. For the gene expression we used the following commercial assays LEP (Hs00174877_m1, RefSeq. NM_000230.2), LPL (Hs00173425_m1, RefSeq. NM_000237.2), SREBF2 (Hs01081784_m1, RefSeq. NM_004599.3), SCD1 (Hs01682761_m1, RefSeq. NM_005063.4), PPARG (Hs01115513_m1, RefSeq. NM_005037.5, NM_015869.4, NM_138711.3, NM_138712.3), IL6 (Hs00174131_m1, RefSeq. NM_000600.4, NM_001318095.1), TNF (Hs01113624_g1, RefSeq. NM_000594.3) and E2F1 (Hs00153451_m1, RefSeq. NM_005225.2). PPIA (4326316E) was used as endogenous control.

### Chromatin immunoprecipitation assay

ChiP was performed as previously described [8]. Briefly, 100 mg of frozen VAT was fixed in 0.5% formaldehyde for 5 minutes and the reaction was stopped by adding a final concentration of 0.125 mM of glycine and incubated for 5 minutes. After homogenizing the sample in cell lysis buffer the nucleus phase was pelleted and then suspended in nucleus lysis buffer [8]. Samples were then sheared by sonication (Bioruptor UCD-300, Diagenode) and the chromatin concentration and chromatin fragments were checked using Nanodrop and 2% agarose gel. Then, samples were immunoprecipitated using an anti-H3K4me3 antibody (ab8580, abcam) linked to magnetic beads (Dynabeads Protein G, Thermofisher) and the DNA purified with MinElute PCR Purification Kit (Qiagen). Specific promoter H3K4me3 was performed using the primer sets depicted in [8] and the following primer set for E2F1 promoter quantification: Sense: AGGAACCGCCGCCGTTGTTCCCG, Antisense: CTGCCTGCAAAGTCCCGGCCACTT. The normalized enrichment value was calculated as the subtraction of the IP relative value with the Input relative value. The IP and Input relative values were calculated by comparing the normalized enrichment values obtained from the standard curves.

### Statistical analyses

Comparisons between groups were calculated using Kruskal-Wallis test for non-normal distribution variables, and Mann-Whitney U-test for the group-by-group comparisons. Spearman’s correlation coefficients were calculated to evaluate the association between the study variables in the whole population. The variable named “MetS variables” defined the number of MetS components present in each individual, which ranged from 0–5, as described by [10]. Values were considered to be statistically significant when P < 0.05. The analyses were performed with SPSS (Version 15.0 for Windows; SPSS).

## Results and discussion

In this work, we studied for the first time the H3K4me3 histone mark at several gene promoters for genes associated with adipogenesis, lipid metabolism and inflammation in human adipose tissue. We identified histone modification (H3K4me3) in a population classified into three groups according to their BMI and glucose metabolic status: Lean NG, MO NG and MO PD. A description of the anthropometric and biochemical variables of these groups is given in Table 1. Statistical differences were observed between groups for BMI, waist circumference and the serum levels of glucose, insulin, HOMA-IR and HDL-C (Table 1).

**Fig 1:**
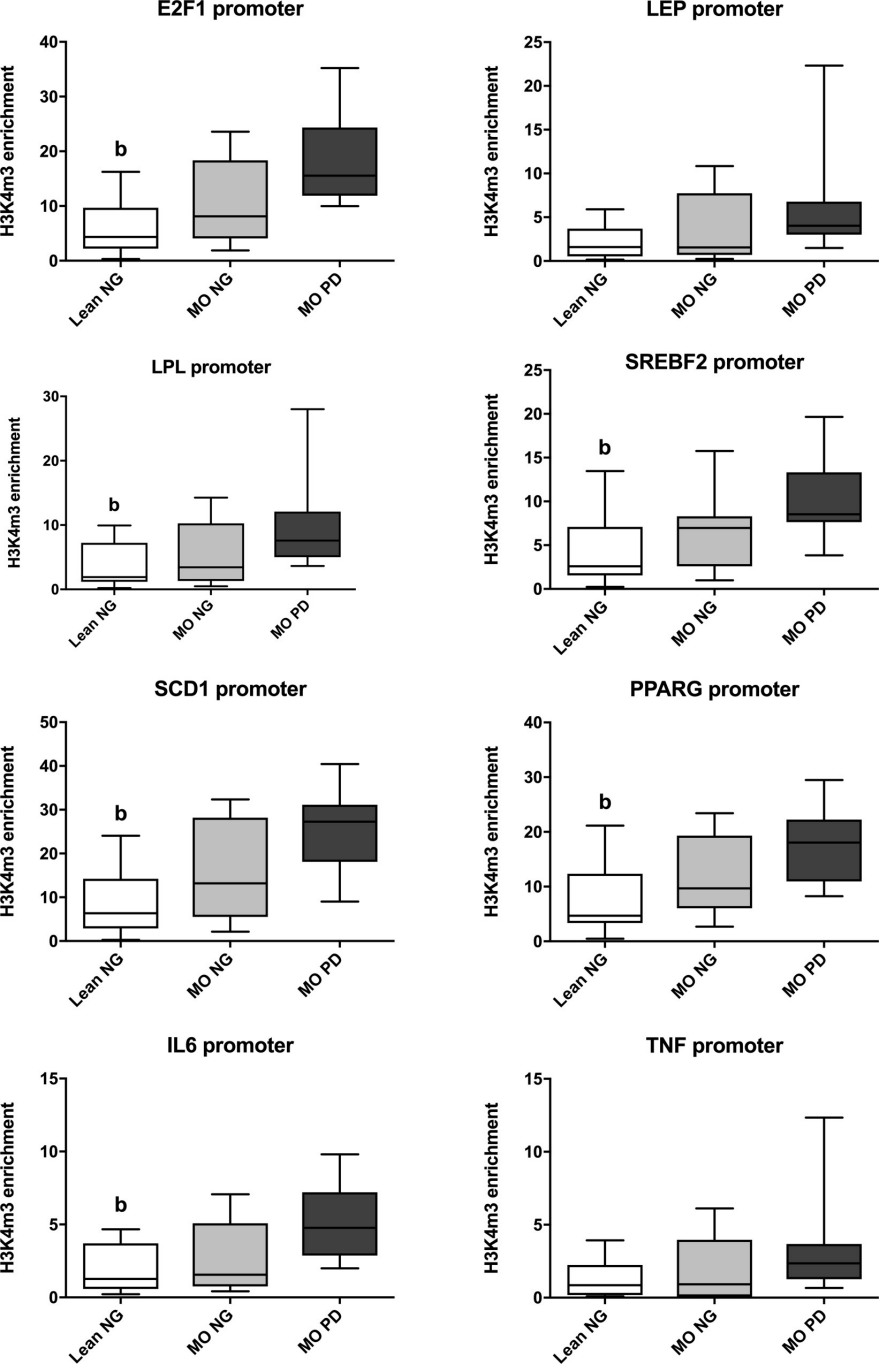
Group comparisons of H3K4me3 mark enrichment at the promoter of the study genes. Different letters indicate significant differences between the means of the different groups of subjects (p < 0.05; a: Lean NG vs. MO NG; b: Lean NG vs. MO PD; c: MO NG vs. MO PD) according to Mann Whitney U test. Abbreviations: Lean Normoglycemic (Lean NG); Morbid obese normoglycemic (MO NG); Morbid obese prediabetic (MO PD); E2F transcription factor 1 (E2F1); Lipoprotein Lipase (LPL); Sterol regulatory element-binding factor 2 (SREBF2); Stearoyl-CoA desaturase 1 (SCD1); Peroxisome proliferator-activated receptor gamma (PPARG); Interleukin 6 (IL6); Tumor necrosis factor (TNF).

**Fig 2:**
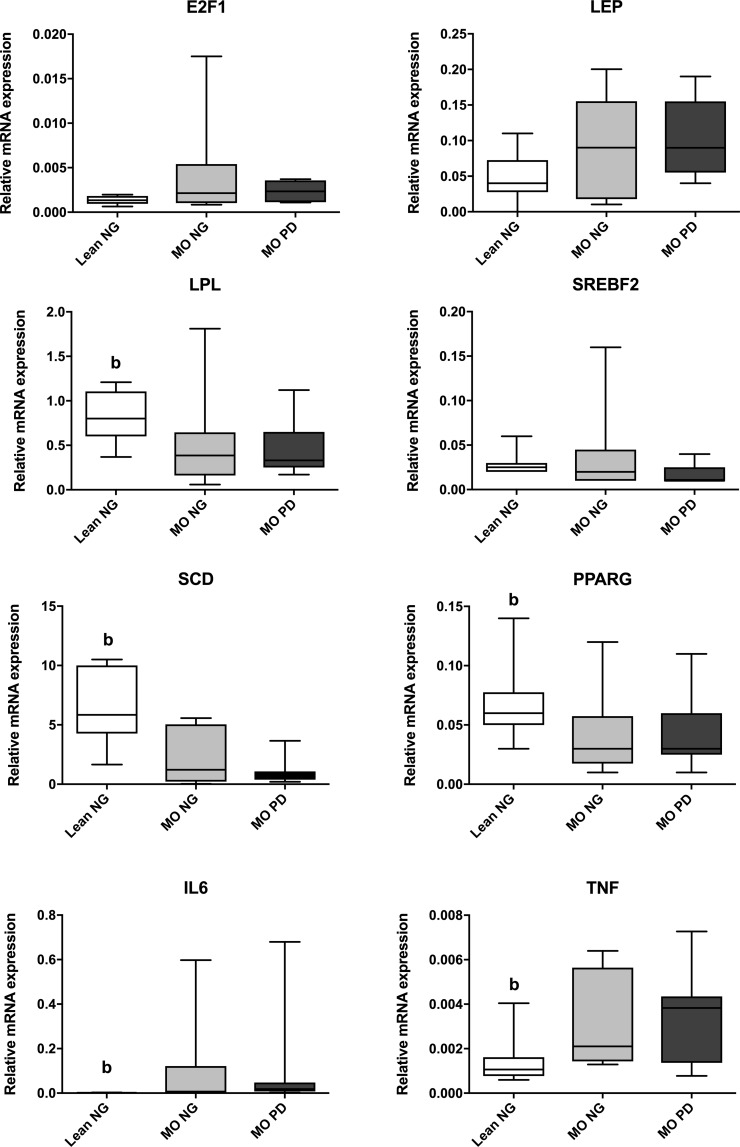
Group comparisons of the relative mRNA levels of the study genes. Different letters indicate significant differences between the means of the different groups of subjects (p < 0.05; a: Lean NG vs. MO NG; b: Lean NG vs. MO PD; c: MO NG vs. MO PD) according to Mann Whitney U test. Abbreviations: Lean Normoglycemic (Lean NG); Morbid obese normoglycemic (MO NG); Morbid obese prediabetic (MO PD); E2F transcription factor 1 (E2F1); Lipoprotein Lipase (LPL); Sterol regulatory element-binding factor 2 (SREBF2); Stearoyl-CoA desaturase 1 (SCD1); Peroxisome proliferator-activated receptor gamma (PPARG); Interleukin 6 (IL6); Tumor necrosis factor (TNF).

Regarding enrichment of the H3K4me3 mark, we observed an increased enrichment at E2F1, LPL, SREBF2, SCD1, PPARG and IL6 promoters in the MO PD group compared to the Lean NG group (Fig 1). Concerning the gene expression, LPL, SCD1 and PPARG mRNA levels were lower in the MO PD group than the Lean NG group (Fig 2); whereas higher mRNA levels for IL6 and TNF genes were noted in MO PD compared to Lean NG subjects (Fig 2).

A positive correlation between the H3K4me3 mark at E2F1, LPL, SREBF2, SCD1, PPARG and IL6 promoters and the BMI and HOMA-IR was observed (Table 2 and S1 Fig). Moreover, there was a positive correlation between glucose and H3K4me3 mark enrichment at the promoter of SCD1, PPARG, E2F1 and IL6 (Table 2 and S1 Fig). The association between the H3K4me3 mark and glucose has been studied in the promoter of the inflammatory gene NFkB p65 in Set7 KD cells, where a decrease of the H3K4me3 mark associated with glucose supplementation was observed [11]. This is in agreement with our findings, showing a positive correlation between promoter H3H4me3 increase and the HOMA-IR. In addition, we also observed a positive correlation between the H3K4me3 mark at E2F1, SREBF2 and SCD promoters and the number of MetS components (MetS Var) present (Table 2 and S1 Fig).

**Table 2:** Spearman correlation analysis between H3K4me3 mark enrichment at the study gene promoters and the anthropometric and biochemical variables. * and ** mean p<0.05 and p<0.01 respectively.

H3K4me3 enrichment	Age	BMI	Glucose	Insulin	HOMA-IR	Tg	Chol	HDL-C	LDL-C	SBP	DBP	MetS Var
**E2F1**	-0.214	0.530**	0.552**	0.573**	0.594**	-0.01	-0.256	-0.163	-0.257	0.123	0.072	0.448*
**LEP**	-0.148	0.364	0.361	0.348	0.367	0.044	-0.133	-0.12	-0.143	0.075	0.006	0.279
**LPL**	-0.168	0.430*	0.395*	0.437*	0.463*	-0.008	-0.208	-0.141	-0.198	0.154	0.064	0.333
**SREBF2**	-0.236	0.467*	0.442*	0.403*	0.441*	-0.048	-0.178	-0.232	-0.113	0.088	0.062	0.463*
**SCD**	-0.242	0.528**	0.513**	0.529**	0.548**	-0.034	-0.214	-0.186	-0.213	0.157	0.071	0.420*
**PPARG**	-0.243	0.488**	0.399*	0.460*	0.479**	-0.051	-0.263	-0.212	-0.246	0.073	0.038	0.369
**IL6**	-0.131	0.430*	0.497**	0.472*	0.501**	0.062	-0.094	-0.15	-0.065	0.191	0.047	0.327
**TNF**	0.009	0.23	0.379*	0.295	0.305	-0.051	-0.093	-0.008	-0.105	0.085	0.056	0.163

E2F transcription factor 1 (E2F1); Leptin (LEP); Lipoprotein Lipase (LPL); Sterol regulatory element-binding factor 2 (SREBF2); Stearoyl-CoA desaturase 1 (SCD1); Peroxisome proliferator-activated receptor gamma (PPARG); Interleukin 6 (IL6); Tumor necrosis factor (TNF); Body mass index (BMI); Homeostatic model assessment of insulin resistance (HOMA-IR); Triglycerides (Tg); Total cholesterol (Chol); High-density lipoprotein cholesterol (HDL-C); Low-density lipoprotein cholesterol (LDL-C); Systolic blood pressure (SBP); Diastolic blood pressure (DBP); Number of MetS variables (MetS Var).

Regarding the correlation between the gene expression and clinical variables, BMI was positively associated with LEP, IL6 and TNF mRNA levels while it was negatively correlated with LPL, SCD1 and PPARG mRNA (Table 3 and S2 Fig). HOMA-IR correlated with LEP, IL6 and TNF mRNA positively, and negatively with SREBF2 and SCD (Table 3 and S2 Fig). These results are in line with the scientific literature, since a negative association between LPL and PPARG mRNA and the BMI has been described previously [9,12]. In addition, BMI and insulin resistance have been associated with a low-grade inflammation in adipose tissue [13–15], which is in accordance with the positive relationship between these two parameters and the inflammatory markers measured in this work. Positive associations were described between HDL-C and LPL, SCD1 and PPARG gene expressions (Table 3 and S2 Fig). Interestingly, E2F1 mRNA levels were negatively associated with all cholesterol variables, total cholesterol (Chol), HDL-C and LDL-C (Table 3). E2F1 has been shown to regulate lipid and cholesterol metabolism in the liver and to participate in cholesterol withdrawal [16,17].

**Table 3:** Spearman correlation analysis between the relative mRNA levels at the study genes and the anthropometric and biochemical variables. * and ** mean p<0.05 and p<0.01 respectively.

Relative mRNA	Age	BMI	Glucose	Insulin	HOMA-IR	Tg	Chol	HDL-C	LDL-C	SBP	DBP	MetS Var
**E2F1**	-0.256	0.359	0.116	0.194	0.19	-0.151	-0.559**	-0.407*	-0.534**	-0.144	0.097	0.284
**LEP**	-0.321	0.522**	0.334	0.673**	0.683**	0.179	-0.213	-0.308	-0.093	0.04	-0.095	0.533**
**LPL**	0.103	-0.500**	-0.295	-0.33	-0.327	-0.189	0.31	0.516**	0.203	0.008	-0.102	-0.454*
**SREBF2**	0.088	-0.258	-0.178	-0.391*	-0.409*	-0.238	-0.082	-0.024	-0.069	-0.209	-0.238	-0.373
**SCD**	0.375*	-0.681**	-0.319	-0.526**	-0.525**	-0.124	0.381*	0.555**	0.277	0.054	-0.165	-0.597**
**PPARG**	0.061	-0.408*	-0.214	-0.216	-0.204	-0.112	0.221	0.371*	0.182	0.097	0.161	-0.28
**IL6**	-0.291	0.729**	0.486*	0.571**	0.590**	0.036	-0.285	-0.223	-0.322	-0.025	0.144	0.533**
**TNF**	-0.298	0.679**	0.322	0.594**	0.612**	0.024	-0.263	-0.175	-0.335	0.048	-0.108	0.444*

E2F transcription factor 1 (E2F1); Leptin (LEP); Lipoprotein Lipase (LPL); Sterol regulatory element-binding factor 2 (SREBF2); Stearoyl-CoA desaturase 1 (SCD1); Peroxisome proliferator-activated receptor gamma (PPARG); Interleukin 6 (IL6); Tumor necrosis factor (TNF); Body mass index (BMI); Homeostatic model assessment of insulin resistance (HOMA-IR); Triglycerides (Tg); Total cholesterol (Chol); High-density lipoprotein cholesterol (HDL-C); Low-density lipoprotein cholesterol (LDL-C); Systolic blood pressure (SBP); Diastolic blood pressure (DBP); Number of MetS variables (MetS Var).

Finally, a harmonized lineal regression analysis showed that BMI was greatly explained by H3K4me3 enrichment levels at the promoter of E2F1 and LPL, and by the mRNA levels of LEP and SCD (Table 4). In this model, these four variables could explain up to 83% of the BMI variability present in our study population (Table 4). Similarly, another harmonized lineal regression analysis (with HOMA-IR as dependent variable) showed that H3K4me3 enrichment levels at the promoter of SCD1 and IL6, together with the mRNA levels of LEP and SCD1, could explain 79% of the variation observed in the HOMA-IR (Table 5).

**Table 4:** Harmonized lineal regression analysis with BMI as dependent variable. H3K4me3 mark enrichment at gene promoters and gene expression of genes that showed significant association in the Spearman correlation analysis were introduced in the model, which was corrected for age and sex.

	BMI (R = 0.91, R^2^ = 0.83)		
	Beta	p	95% CI
E2F1 H3K4me3	0.979	0.001	0.844 to 2.922
LPL H3K4me3	-0.813	0.003	-3.640 to -0.840
LEP mRNA	0.344	0.002	38.32 to 152.97
SCD mRNA	-0.516	0.000	-3.650 to -1.459

Body mass index (BMI); E2F transcription factor 1 (E2F1); Lipoprotein Lipase (LPL); Leptin (LEP); Stearoyl-CoA desaturase 1 (SCD1).

**Table 5:** Harmonized lineal regression analysis with HOMA-IR as dependent variable. H3K4me3 mark enrichment at gene promoters and gene expression of genes that showed significant association in the Spearman correlation analysis were introduced in the model, which was corrected for age and sex.

	HOMA-IR (R = 0.89, R^2^ = 0.79)		
	Beta	p	95% CI
Gender	-0.259	0.047	-3.598 to -0.027
SCD H3K4me3	0.792	0.016	0.049 to 0.417
IL6 H3K4me3	-0.666	0.030	-1.769 to -0.105
LEP mRNA	0.564	0.000	16.60 to 44.82
SCD mRNA	-0.261	0.065	-0.541 to 0.018

Homeostatic model assessment of insulin resistance (HOMA-IR); Stearoyl-CoA desaturase 1 (SCD1); Interleukin 6 (IL6); Leptin (LEP).

The H3K4me3 level depends on the methylation and de-methylation processes, which are carried out by several methyltransferases and de-methyltransferases [7], and that in turn depend on substrate disposal [18]. S-Adenosyl methionine (SAM) is a methyl-group molecular donor for a wide range of reactions, including methylation processes and has been shown to be altered according to nutritional and cell metabolism status affecting H3K4me3 levels [19]. It has been shown that SAM levels increase with the BMI, and that this increase is associated with adiposity [20]. Therefore, a possible increase in SAM associated with the BMI could explain the increase in the H3K4me3 mark found in E2F1, LPL, SREBF2, SCD1, PPARG and IL6 gene promoters of the MO PD group with respect to the Lean NG group. Moreover, in our population the differences in the H3K4me3 enrichment levels for the genes associated with BMI and HOMA-IR might be related to substrate fluctuations, even though it has been shown that sensitivity of histone methylation to methyl group availability is site-specific [18]. Accordingly, the fluctuation in the H3K4me3 mark observed in this study could be related with the metabolic status since energy and metabolic intermediaries have been related to histone methylation, which could be in tune with adipose tissue dysfunction.

De-methylation processes can also modulate H3K4me3 mark levels. The reduction in fatty acid oxidation and glucose incorporation observed in insulin resistance states [21–23] together with the adipose tissue hypoxia in the context of metabolic diseases and insulin resistance [24] could affect H3K4me3 mark levels by down-regulating histone de-methylation. In fact, hypoxia has been reported to increase H3K4me3 marks through reduction of de-methylation [18].

In order to analyze whether the promoter H3K4me3 mark levels could be related to gene expression we performed Spearman’s correlation analysis in the whole population between the mRNA levels and H3K4me3 mark at each gene. However, we did not observe any significant association between the promoter H3K4me3 levels and the mRNA levels for any gene excepting E2F1, in which a positive correlation was observed (r = 0.422, p = 0.04). H3K4me3 is a mark associated with a condition of open chromatin and consequently stimulates gene expression [25]. Thus, this positive association observed between this mark at the promoter of E2F1 and the mRNA levels for this gene suggests that this gene could be transcriptionally controlled by this epigenetic mark in adipose tissue. Adipose tissue E2F1 has been shown to be overexpressed in obesity, leading to inflammation and metabolic deterioration [26,27]. Thus, the H3K4me3 mark level might depend on the metabolic status of the patients, and the possible increase of this mark at E2F1 promoter could in turn be related to the worsening in the metabolic status through direct stimulation of E2F1 expression. With respect to the other genes studied, further studies will be necessary to explain their complex epigenetic regulation, for instance through the study together with other histone marks like H3K27m3 (related to heterochromatin and gene repression), the study of DNA methylation at their promoter or ChIP followed by deep sequencing (ChIPseq), which could highlight a more accurate regulatory state of the promoters [28,29]. Other authors have previously described that there are expressed genes where the mark is absent and genes with the mark that are not expressed, showing that the presence of the mark is neither necessary nor sufficient for gene expression even though genes marked with H3K4me3 have a higher average levels of mRNA [30].

Nevertheless, chromosome conformation is important for the genetic regulation in adipocytes [31], a process that might be influenced by H3K4me3 mark modifications [32,33]. Chromosome deregulation has been widely associated with several diseases, some associated with H3K4me3 methylases or H3K4me3 modifier deregulation [7]. In fact, abnormal H3K4me3 marks in brain tissue have been observed in the context of Huntington’s disease without the existence of a correlation between the difference in H3K4me3 peaks observed with their associated gene expression [34], similar to our results in VAT. In this sense, histone modifications could not only be important for gene expression regulation but also for chromosome stability, DNA replication or chromosome segregation [35].

However, the cross-sectional nature of our study represents a limitation and more studies using ChiP-seq, metabolomic approaches and animal models are necessary to establish the role of the H3K4me3 mark in VAT and how the metabolic status could be affecting H3K4me3 (and other histone marks) levels.

As strengths, in this work we study for the first time H3k4me3 after ChIP in human adipose tissue, a metabolic tissue in which histone characterization is yet to be performed due to its lipid nature which hinders downstream manipulation [8].

In conclusion, we applied for the first time the adipose tissue ChIP protocol to determine the histone H3K4 trimethylation modification at the promoter of several adipogenic, lipid metabolism and inflammatory genes in human VAT in a population with different degrees of BMI and glucose metabolism. We found an increase in the H3K4me3 mark at E2F1, LPL, SREBF2, SCD1, PPARγ and IL6 promoters as the BMI and HOMA-IR increased. Moreover, enrichment at these promoters was positively associated with BMI, HOMA-IR and MetS worsening. Interestingly, E2F1 mRNA expression and the H3K4me3 mark level correlated positively, suggesting a transcriptional control of this gene by this histone modification in VAT, contributing to VAT inflammation and deterioration in obesity and metabolic diseases. Thus, this work points out the importance of epigenetics in discerning complex metabolic diseases and obesity, and gives clues to a more comprehensive and in depth analysis about the possible role of adipose tissue histone code within the context of these disorders, which could lead to possible epigenetic therapies.

## Supporting information

S1 Fig Scatterplot showing the significant associations between H3K4me3 enrichment at the study genes and the metabolic parameters. (DOCX)

S2 Fig Scatterplot showing the significant associations between mRNA levels at the study genes and the metabolic parameters. (DOCX)

## Abbreviations

BMI: Body mass index
ChIP: Chromatin immunoprecipitation
E2F1: E2F transcription factor 1
ELISA: Enzyme immunoassay
H3K4me3: H3K4 trimethylation
IL6: Interleukin 6
Lean NG: Lean normoglycemicl
LEP: Leptin
LPL: Lipoprotein Lipase
MetS Var: Metabolic syndrome components
MetS: Metabolic syndrome
MO DB: Morbid Obese Diabetic
MO NG: Morbid Obese Normoglycemic
MO PD: Morbid Obese Prediabetic
PPARG: Peroxisome proliferator-activated receptor gamma
SAH: S-Adenosyl Homocystein
SAM: S-Adenosyl methionine
SCD1: Stearoyl-CoA desaturase 1
SREBF2: Sterol regulatory element-binding factor 2
TCA: tri-carboxylic acid cycle
TG: triglycerides
TNF: Tumor necrosis factor
VAT: visceral adipose tissue
