# Supplementary material for: Human adipose tissue H3K4me3 histone mark in adipogenic, lipid metabolism and inflammatory genes is positively associated with BMI and HOMA-IR
Source: PLoS One. 2019 Apr 8;14(4):e0215083. doi: 10.1371/journal.pone.0215083 (PMC6453466; doi:10.1371/journal.pone.0215083)
Supplement: S1 Fig — (DOCX) [file pone.0215083.s001.docx]

**S1 Figure**. Scatterplot showing the significant associations between H3K4me3 enrichment at the study genes and the metabolic parameters.
